# Misdiagnosis of pancreatic papillary mucinous cystadenocarcinoma: A case report

**DOI:** 10.3892/ol.2014.2242

**Published:** 2014-06-12

**Authors:** PENG-FEI QIAO, GUANG-MING NIU, YANG GAO

**Affiliations:** Department of Magentic Resonance Imaging, Affiliated Hospital of Inner Mongolia Medical University, Hohhot, Inner Mongolia 010050, P.R. China

**Keywords:** papillary cystadenocarcinoma of the pancreas, misdiagnosis, spinal metastasis

## Abstract

The morbidity of papillary cystadenocarcinoma of the pancreas is extremely low and the condition is rarely first found as spinal metastases, thus it is often misdiagnosed prior to surgery. The present study reports a case of papillary cystadenocarcinoma with thoracolumbar metastases in a 56-year-old male. The first symptom to occur was backache, however, computed tomography revealed no positive findings. The pain became exacerbated and the patient underwent lumbar and thoracic vertebrae magnetic resonance imaging, which identified abnormal signals. Imaging and pathological examinations were used for the final diagnosis. Due to multiple bone metastases, the patient the administration of induction chemotherapy was suggested, however, the patient refused. The patient succumbed to the disease in June 2013.

## Introduction

Adenocarcinoma is a type of tumor that originates from the glandular epithelial cells. When it secretes more mucous it is termed mucinous carcinoma. Mucinous carcinomas that have a large number of papillary structures termed papillary adenocarcinomas, and cystic carcinomas that have highly expanded lumina are known as cystadenocarcinomas (1). Cystadenocarcinomas may be accompanied by papillary growth, and are then termed papillary cystadenocarcinomas (2). Papillary cystadenocarcinomas usually occur in the pancreas, with an incidence rate of <1% of all pancreatic tumor cases. The primary clinical manifestation of papillary cystadenocarcinoma is abdominal distension or pain, as well as occasional obstructive jaundice (3,4). The carcinoma is a potentially low-grade malignant tumor. Currently, radical surgical resection is the only effective treatment, and a correct diagnosis is dependent on histopathological examination (5). Patient provided written informed consent.

## Case report

### Patient presentation

**Figure 1:**
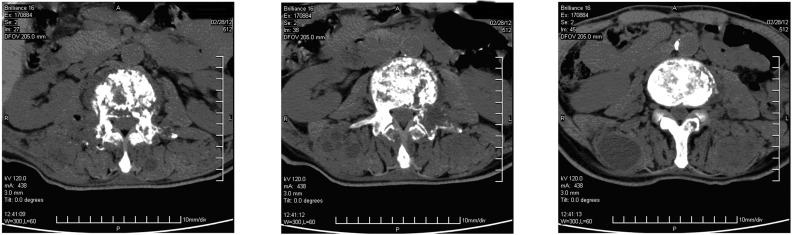
Computed tomography (CT) scan showing multiple myelomata. Chest 12/waist 1 vertebral level spinal stenosis.

A 56-year old male patient presented with backache that had persisted for more than seven months, with exacerbation of the pain for half a month. Six months prior to this, the patient felt pain in the back with no clear predisposing cause. In October 2011, the patient sought medical advice from the Affiliated Hospital of Inner Mongolia Medical University (Hohhot, China). A lumbar computed tomography (CT) scan revealed a lumbar disc bulging between the third and fourth lumbar vertebrae. The patient received Chinese medicinal pills (dosage and composition unknown) and plaster external treatment; however, the results were not effective. In January 2012, half a month prior to the current admittance to hospital, the patient felt lumbar acid, with exacerbated pain, weakness of the double lower limbs, activity limitations and dark brown urine. The patient consulted The Second Affiliated Hospital of Inner Mongolia Medical College (Hohhot, China), and CT revealed some specific findings (Fig. 1).

In February 2012, in order for further diagnosis and treatment, the patient was hospitalized in the Department of Orthopedics of the Affiliated Hospital of Inner Mongolia Medical University. Since the onset of the symptoms, the patient had experienced alternating diarrhea and constipation, and had lost ~30 pounds in weight. The patient’s family history was not contributory, and physical and library examinations revealed no abnormalities.

### Diagnosis

**Figure 2:**
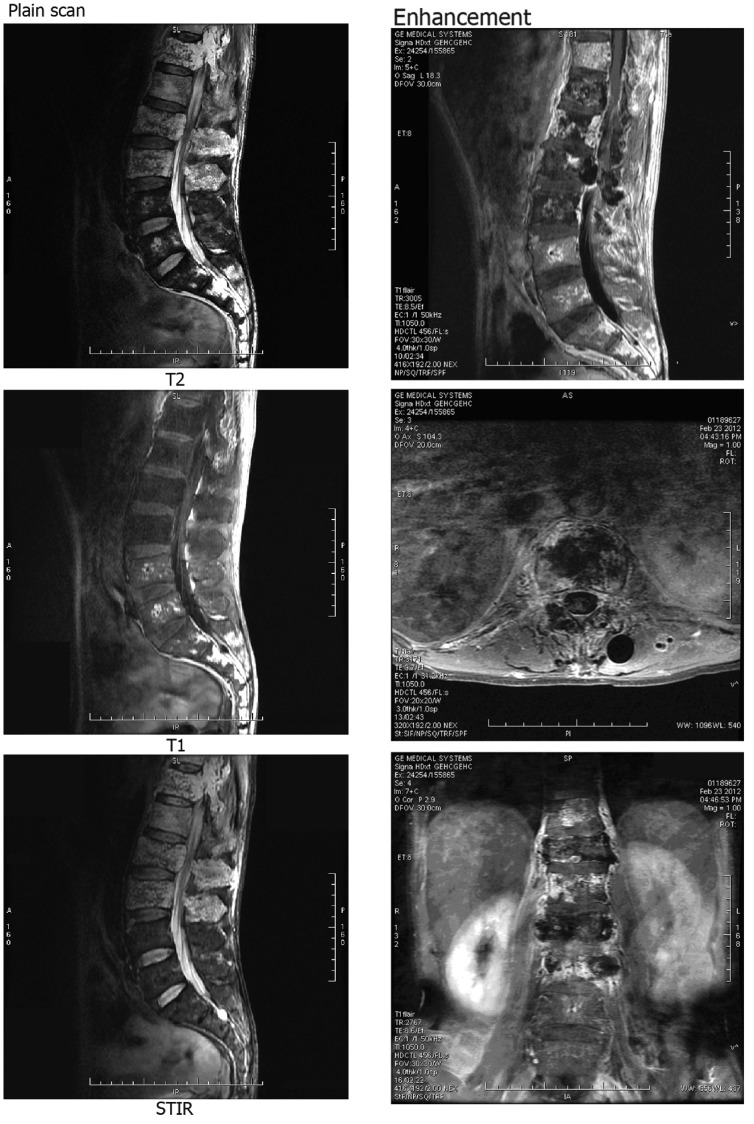
Lumbar vertebrae magnetic resonance imaging (MRI) showing abnormal signals in chest 12-waist 3 vertebral body and spine, with long T1 and long T2 signals. The signal was uneven and the corresponding level back muscle and soft tissue showed mixed T1 and T2 signals, with multiple cystic long T1 and T2 signals. The disc structure appeared normal and the perturbation of the spinal cord and cauda equinal nerve coursed naturally with no abnormal signal in the spinal cord. Enhanced scanning revealed that the lesion exhibited inhomogeneous enhancement and the cystic lesions showed ring enhancement. STIR, short T1 inversion recovery.

**Figure 3:**
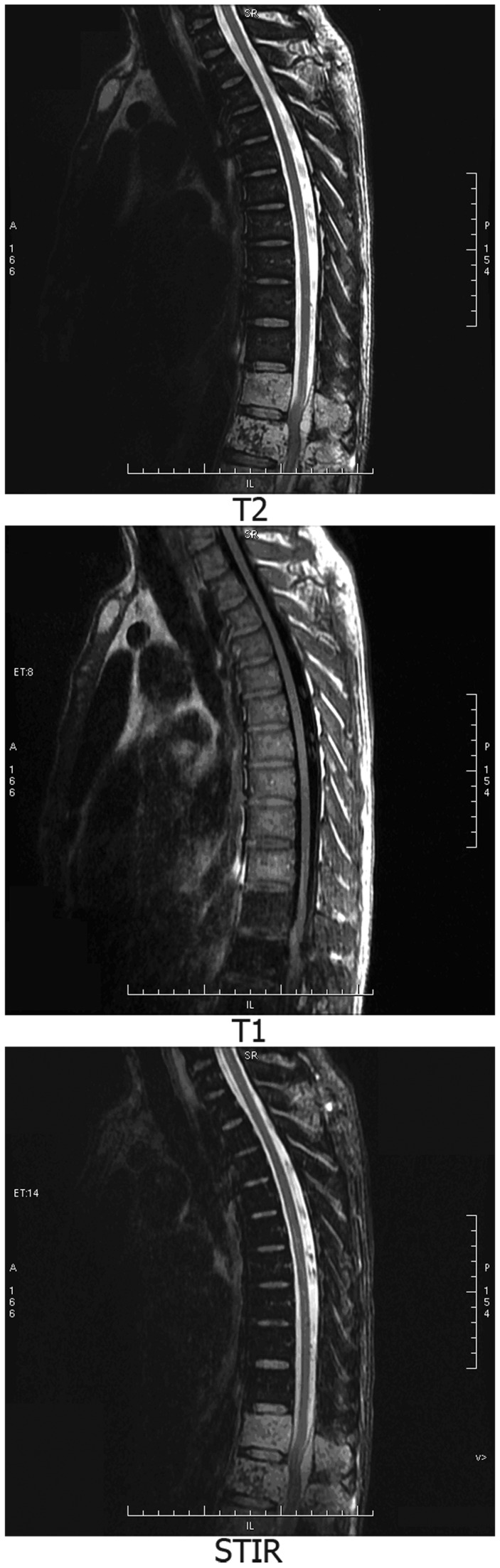
Thoracic vertebrae magnetic resonance imaging (MRI) scan showing abnormal signals in the 3rd and 4th thoracic spinous processes and the posterior soft tissues. STIR, short T1 inversion recovery.

**Figure 4:**
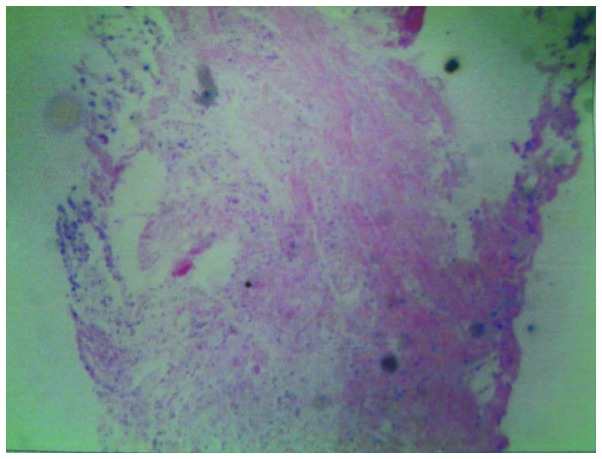
The fibrous connective tissue and bone tissue of the second lumbar vertebrae and paravertebrae in the papillary mucinous epithelial cyst.

Lumbar vertebrae magnetic resonance imaging (MRI) (Fig. 2) and thoracic MRI (Fig. 3) scans showed abnormal signals. The CT and MRI findings revealed multiple myeloma and multiple bone metastases. A Bence-Jones protein test and biopsy were performed, with negative results. However, the pathological diagnosis was positive (Fig. 4). Based on these findings and the patient history, the patient was diagnosed with malignant papillary mucinous cystadenocarcinoma.

**Figure 5:**
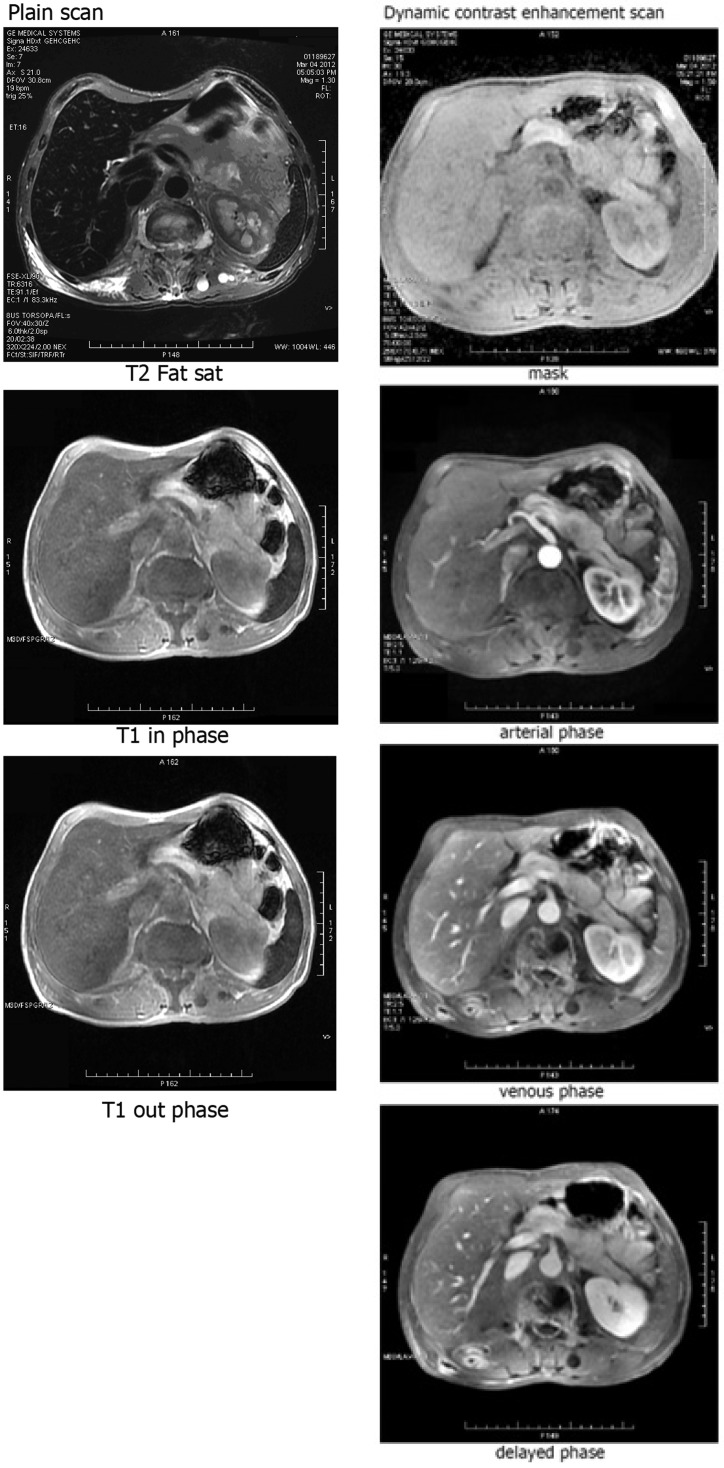
Abdominal magnetic resonance imaging (MRI) scan showing a 2.1×1.8-cm mass with mixed T1 and mixed T2 abnormal signals in the body of the pancreas, with circular-like and peripheral edema. Neighboring tissues were compressed. Enhanced MRI scans showed that the mass was unevenly enhanced.

When the imaging and pathological diagnoses were combined, the source of the spinal multiple malignant tumors was not clear. Malignant papillary mucinous cystadenocarcinoma is primarily derived from glands, including those of the respiratory and gastrointestinal tracts. Based on analyses of the respiratory and digestive tracts, and other laboratory examinations and imaging, the tumor was located in the body of the pancreas (Fig. 5).

### Patient outcome

In June 2013, the patient succumbed to the condition. Retrospective analyses suggested that if the pancreatic lesions had been detected and treated earlier, the prognosis may have been improved.

## Discussion

Solid cystic papillary tumor of the pancreas (SCPT) is a rare form of pancreatic cancer and was first reported by Frantz (4) in 1959. The disease is also confusingly known as pancreatic solid and papillary tumor, pancreatic solid and papillary epithelial tumor, pancreatic papillary cystic solid tumor, pancreatic papillary adenoma/carcinoma and pancreatic solid cystic papillary tumor (6,7). The World Health Organization’s tumor histological classification of 2004 classified the tumor as a solid false papilloma; listed as a source of pancreatic exocrine tumors (8).

SCPT often occurs in the pancreatic tail, its growth is relatively slow and the clinical symptoms are insidious. The tumors are round or oval in shape, with a clear boundary and a fibrous capsule. SCPT has sections with the structure of solid and cystic lesions, and is often is full of blood or gelatin. Under light microscopy, the tumor cells appear consistent, with clear or eosinophilic cytoplasm, round or oval nuclei, no obvious atypia and with low numbers of mitotic figures. The tumor cells around the vascular complex layer are arranged in papillary and solid areas alternately, with different degrees of visible hemorrhage and cystic change (9).

The origin of SCPT is controversial, as the tumor can derived from outside or within the pancreas (10). Immunohistochemical studies have reported the diversification of the tumor and have no specific findings. SCPT has been reported to exhibit positive staining for neuron-specific enolase (84.1%), antitrypsin (83.1%) and vimentin (72.1%), and the positive rate of progesterone and estrogen receptor expression is 35.1 and 5.1%, respectively (11).

It is difficult to distinguish between the imaging findings from papillary cystadenoma and cystadenocarcinoma (12). However, cystadenomas often exhibit a clear boundary around the circular or oval cystic lesions and do not generally involve the main pancreatic duct. The cyst fluid has a low density on CT, high signal on T2-weighted imaging (T2WI) and generally low signal on T1-weighted imaging (T1WI), with no enhancement on dynamic enhanced scans. The cystic wall and septum have soft tissue density on CT, occasionally with strip or curved calcification. On MRI, the cystic wall and septum have relatively low signals on T2WI, soft tissue signals on T1WI and mild enhancement on enhanced scans. The mural nodules attached to the wall or gap, have soft tissue density and signals, or mild to severe enhancement on enhanced scans. Furthermore, the more solid components are more likely to be borderline of malignant tumors. Therefore, certain studies have hypothesized that cystadenoma and cystadenocarcinoma reflect different stages of lesion development (13). In the present case, the patient initially presented with backache and lumbar discomfort, with no bone destruction or other positive signs on CT. The doctors were not familiar with the disease, therefore, it was difficult to make a correct diagnosis, delaying the treatment.

Surgical resection, including local tumor resection, resection of the pancreatic body and tail and pancreatectomy, is the primary method for treating SCPT. SCPT has a good prognosis and survival period, with a two-year survival rate of 97% and a five-year survival rate of 95% (14).

In conclusion, due to the low incidence and difficult diagnosis of SCPT, as well as the lack of specific laboratory and imaging examinations, doctors should be aware of the possibility of SCPT when the location or imaging features are not typical, particularly if the first symptoms are metastases.
